# Supplementary material for: Prevalence and Prognostic Role of IDH Mutations in Acute Myeloid Leukemia: Results of the GIMEMA AML1516 Protocol
Source: Cancers (Basel). 2022 Jun 18;14(12):3012. doi: 10.3390/cancers14123012 (PMC9221405; doi:10.3390/cancers14123012)
Supplement: Supplementary file 1 [file cancers-14-03012-s001.zip › cancers-1747285-supplementary.pdf]

Table S1. Univariate Logistic regression model for CR.

Characteristic	OR [1]	95% CI [1]	*p*-value
**Gender**			
M	—	—	
F	1.65	0.97, 2.81	0.065
**Age starting treatment**	0.96	0.94, 0.98	**<0.001**
**WBC**	1.00	1.00, 1.01	0.23
**HB**	1.06	0.93, 1.23	0.40
**PLTS**	1.00	1.00, 1.00	0.50
**Blasts**	1.00	0.99, 1.01	0.54
**WHO PS**			
0	—	—	
I	0.73	0.40, 1.32	0.29
II	0.20	0.07, 0.51	**0.001**
III	0.41	0.08, 1.97	0.26
**AML type**			
de novo	—	—	
secondary	0.17	0.06, 0.39	**<0.001**
therapy related	0.59	0.19, 1.80	0.35
**AML secondary**			
MDS	—	—	
PV	0.00		>0.99
MF	1.90	0.08, 24.3	0.63
FLT3			
wild type	—	—	
ITD	1.06	0.53, 2.12	0.88
TKD	5.78	1.00, 109	0.10
ITD & TKD	0.00		0.99
NPM1			
wild type	—	—	
mutated	2.26	1.20, 4.42	**0.013**
TP53			
wild type	—	—	
mutated	0.00		>0.99
CEBPA			
wild type	—	—	
mutated	28,363,208	0.00, NA	>0.99
IDH1			
wild type	—	—	
mutated	1.34	0.64, 2.89	0.44
IDH2			
wild type	—	—	
mutated	1.19	0.60, 2.37	0.62
IDH1/IDH2			
IDH1 IDH2 WT	—	—	
IDH1 mutated	1.38	0.65, 3.02	0.41
IDH2 mutated	1.26	0.63, 2.55	0.52
IDH1/2			
IDH1/2 WT	—	—	
IDH1/2 mutated	1.31	0.75, 2.30	0.35
**Karyotype**			

**Characteristic**	**OR [1]**	**95% CI [1]**	***p*-value**
other karyotype	—	—	
complex karyotype	0.46	0.16, 1.22	0.12
**Treatment**			
Conventional CHT	—	—	
Hypomethylating	0.28	0.13, 0.57	**<0.001**

[1] OR = Odds ratios CI = confidence interval.
